# Energy Level Prediction of Organic Semiconductors for Photodetectors and Mining of a Photovoltaic Database to Search for New Building Units

**DOI:** 10.3390/molecules28031240

**Published:** 2023-01-27

**Authors:** Jehad Saleh, Sajjad Haider, Muhammad Saeed Akhtar, Muhammad Saqib, Muqadas Javed, Sayed Elshahat, Ghulam Mustafa Kamal

**Affiliations:** 1 Chemical Engineering Department, College of Engineering, King Saud University, P.O. Box 800, Riyadh 11421, Saudi Arabia; 2 School of Chemical Engineering, Yeungnam University, Gyeongsan 38541, Republic of Korea; 3 Institute of Chemistry, Khwaja Fareed University of Engineering & Information Technology, Rahim Yar Khan 64200, Pakistan; 4 Physics Department, Faculty of Science, Assiut University, Assiut 71516, Egypt

**Keywords:** machine learning, energy levels prediction, semiconductor photodetectors, regression models, pearson correlations

## Abstract

Due to the large versatility in organic semiconductors, selecting a suitable (organic semiconductor) material for photodetectors is a challenging task. Integrating computer science and artificial intelligence with conventional methods in optimization and material synthesis can guide experimental researchers to develop, design, predict and discover high-performance materials for photodetectors. To find high-performance organic semiconductor materials for photodetectors, it is crucial to establish a relationship between photovoltaic properties and chemical structures before performing synthetic procedures in laboratories. Moreover, the fast prediction of energy levels is desirable for designing better organic semiconductor photodetectors. Herein, we first collected large sets of data containing photovoltaic properties of organic semiconductor photodetectors reported in the literature. In addition, molecular descriptors that make it easy and fast to predict the required properties were used to train machine learning models. Power conversion efficiency and energy levels were also predicted. Multiple models were trained using experimental data. The light gradient boosting machine (LGBM) regression model and Hist gradient booting regression model are the best models. The best models were further tuned to achieve better prediction ability. The reliability of our designed approach was further verified by mining the photovoltaic database to search for new building units. The results revealed that good consistency is obtained between experimental outcomes and model predictions, indicating that machine learning is a powerful approach to predict the properties of photodetectors, which can facilitate their rapid development in various fields.

## 1. Introduction

The world is a place of discovery and billions of devices containing multiple sensors have been commercialized. Photodetectors or photosensors primarily work as optical receivers for the conversion of light into electrical signals. The photodetector has become a vital part of modern devices with a broad range of applications, including environmental monitoring, optical communication, health monitoring, image sensing, defense system and for safety purposes in industries [1]. In the modern age, silicon (Si), germanium (Ge), and indium gallium arsenide (InGaAs)-based inorganic photodetectors (PDs) have been popular in the market due to their stable performance, high quantum efficiency, high sensitivity/detectivity, response speed or responsivity. Despite having several advantages, inorganic photodetectors have limitations that cannot be ignored, such as complex fabrication procedures, manufacturing cost (e.g., requirement of high vacuum environment, high processing temperature and complex growth) and brittleness [2,3]. Moreover, inorganic photodetectors are rigid in nature, which limits their utilization in flexible environments or applications [4,5].

In recent years, organic photodetectors have emerged as a promising alternative for inorganic photodetectors [6]. These show various superiorities, including operation with a cooling-free effect, compatibility with flexible devices, detectable spectral response, and easy processing, which makes them potential candidates in wearable electronics. The spectral response can be achieved by altering the material used for organic semiconductors in the case of organic photodetectors (OPDs) [7,8]. In comparison, to inorganic semiconductors, organic semiconductors are composed of carbon-based molecules, which makes the organic photodetectors more environmentally friendly and biocompatible. In addition, various polymers and small molecules have been used for organic semiconductors [9,10]. These compounds generate a positive impact on electronic and optoelectronic devices [11]. Much progress has been made in recent years in the development of organic semiconductor devices [12].

A narrow bandgap is required for high-performance organic semiconductor photodetectors [13,14,15]. For decades, semiconductors with a large bandgap have been utilized. When the light of high energy falls on the target material, the excitons generated in donor front layers are unable to separate into free charges properly. Only low-energy photons with the power of long penetration depth can reach the donor-acceptor interface and then successfully generate the free charge. Therefore, it is an area of ongoing interest to predict energy levels of organic semiconductors for photodetectors and mining of the photovoltaic database to search for new building units. Traditionally, new designs can be achieved by utilizing the knowledge gained from laborious and multistep synthesis procedures, expensive device optimization and characterization. However, these trial-and-error methods do not guarantee success in the end. Moreover, it is hard to predict the performance of the materials before performing expensive experimental work. To this end, computer-aided material designing, discovery, and screening are of utmost importance.

Computational science is a popular field of science, which can be effectively applied to solve the complex problems of various systems and to finally find the solutions for such scientific problems [16,17,18]. Computational methods can analyze, screen, and predict data through mathematical algorithms. These methods can be applied to various fields of science [19,20,21,22]. During the past decade, researchers have been focusing on developing predictive computer models. These models can help them to analyze challenging problems [23]. Machine learning is a modern research tool [24]. Machine learning analysis (MLA) is based on pattern recognition by reducing the size of data and those parameters which can be learned by the computer. In machine learning, the results can be obtained by analyzing previously reported studies [25]. Moreover, several properties can be studied without understanding the chemistry or physics behind these properties [26]. The recent advancement in MLA includes the successful prediction of properties of the materials, materials discovery, drug development and material designing. Molecular fingerprinting and similarity analysis is now a common feature. Various algorithms can be used to train the models to obtain good accuracy. Machine learning can be used to predict the values of the highest-occupied molecular orbital (HOMO) and lowest-unoccupied molecular orbital (LUMO and power conversion efficiency (PCE) with accuracy. In recent studies, MLA has been used to design efficient molecules for organic photovoltaic (OPV) applications, and the structures of these molecules pass through subsequent successful experimental testing. Efficient organic semiconductor materials have been designed by using this approach, which accelerates the developing process in a time-saving manner [27].

Herein, a machine learning-based approach was applied to predict the energy level of organic semiconductors for photodetectors. Multiple models were trained, and their respective parameters were adjusted. As a result, the models with the highest accuracy were chosen for conducting further analyses. Moreover, detailed data about energy levels (HOMO and LUMO) were visualized to show the trend (hidden pattern) in the data. The parameter’s feature importance was also evaluated for training machine learning models. In addition, Pearson correlation and Shapiro ranking was applied to demonstrate the correlation between different parameters. A similarity analysis was performed to find the similarities between reference structure and structure in the database. Furthermore, mining of the photovoltaic database was used to search for new building units.

## 2. Results and Discussions

The performance of varied materials depends on their chemistry [28,29,30]. Chemical data can help to understand their behavior [31,32]. The hidden pattern of data can provide much useful information [33].

### 2.1. Molecular Descriptors

Molecular descriptors are the mathematical representation of the molecules used to train the models based on machine learning (Table 1) [34,35,36]. Molecular descriptors can be generated with the help of different algorithms. These can be derived from the chemical structure of the molecules. Physical and chemical properties or information can be described quantitatively with the help of the numerical value of molecular descriptors [37,38]. Almost thousands of molecular descriptors were calculated. Then, these were shortlisted in several unique ways. Molecular descriptors are based on independent properties. They can help researchers to perform similarity tests by using different models such as RDkit in molecular libraries. Based on the similarities present in the descriptor values, the molecules with the same physical and chemical properties can be evaluated.

### 2.2. Regression Analysis

The performance of machine learning is strongly dependent on algorithms [39]. To identify which kind of variable has a strong effect on the topic of interest, these methods are effectively reliable. The regression analysis provides information on the way these factors influence each other, how to determine the factors that are of most importance, and which factors can be ignored. Regression analysis uses various algorithms of machine learning. The data can be integrated into two parts: the testing set and the training set. These data are of different ratios, 70% 30%, 60% 40%. The best one is training: test ratio. Afterwards, by analyzing the values of predicted PCE and experimental PCE, the correlation between these two was calculated. The obtained results are plotted in the form of a graph.

### 2.3. Pearson Ranking Correlation

In machine learning algorithms, Pearson correlation is the most widely used correlation for numerical variables. To effectively measure the degree of relationship (linear association or correlation) between two different variables, this correlation can be used. It shows how far the data points are from the line of best fit. For this method to work effectively, the variables should be normally distributed. The direction and strength of two variables can be measured by the number between 1 and −1, where −1 indicates negative correlation, 1 represents positive correlation, and 0 indicates no correlation.

### 2.4. HOMO Prediction

**Figure 1:**
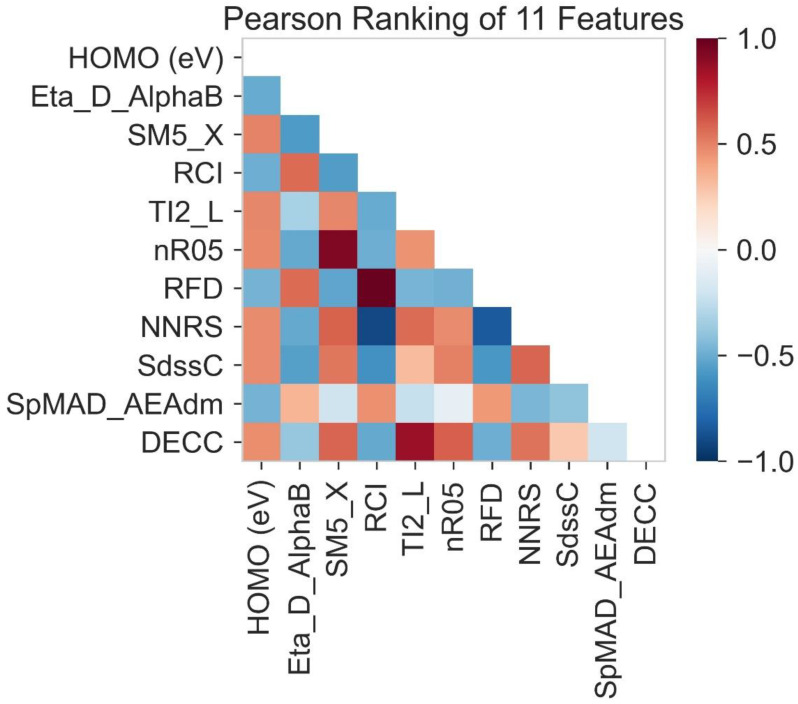
Correlation between HOMO and molecular descriptors.

The value from 1 to 0 shows a positive correlation between the HOMO and other molecular descriptors. The value from 0 to −1 shows the negative correlation between HOMO and other molecular descriptors. The 0 value shows no correlation between the variables. The red color indicates a positive correlation, while blue color indicates a negative correlation. As shown in Figure 1, DEEC, SdssC, NNRS, Nr05, ti2-L, and SM5-X all are red in color, these molecular descriptors show a positive correlation with the HOMO lies on the x-axis. In contrast, SPMAD-AEAdm, RFD, RCI and Eta-D-AlphaB show a negative correlation with the HOMO of the *x*-axis. In addition, TI2-L shows a strong positive correlation with the HOMO lying on the *y*-axis.

**Figure 2:**
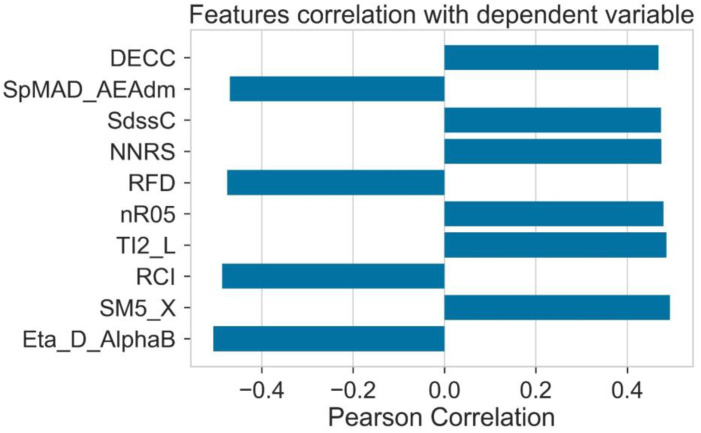
Correlation of features with HOMO.

For model training, calculated molecular descriptors are used as a source of input. The chemistry of donor molecules is represented by the help of molecular descriptors. The numerical form is used for presenting the chemical structure of materials in the numerical form presented by the molecular descriptors. Figure 2 shows that the Eta-D-AlphaB, RCI, RFD, and SPMAD-AEAdm show the negative dependent Pearson correlation. On the other hand, other molecular descriptors such as DECC, SdssC, NNRS, Nr05, TI2-L, and SM5-X show that they are positively dependent features.

### 2.5. Shapiro Ranking

**Figure 3:**
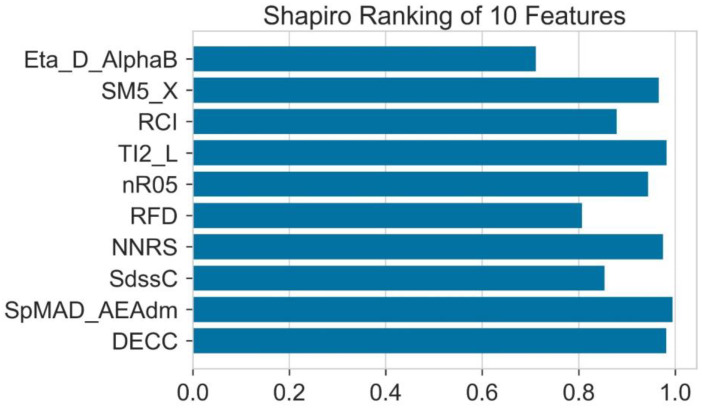
The normality of all the features analyzed by Shapiro test.

The Shapiro ranking test is also known as the Shapiro–Wilk test. This test is usually performed to test the normality in statistics. Martin Wilk and Samuel Sanford Shapiro published this in 1965. This ranking uses the Shapiro–Wilk algorithm, which is generated by the Yellowbrick Python package. This is a one-dimensional feature ranking. To assess the normality of distribution, it considers a single feature at a time. The results are shown in the form of a bar plot, which shows the features with the maximum score on the one side and the features using the average score on the other side. Figure 3 shows that Eta-D-AlphaB shows the least amount of distribution according to this ranking.

**Figure 4:**
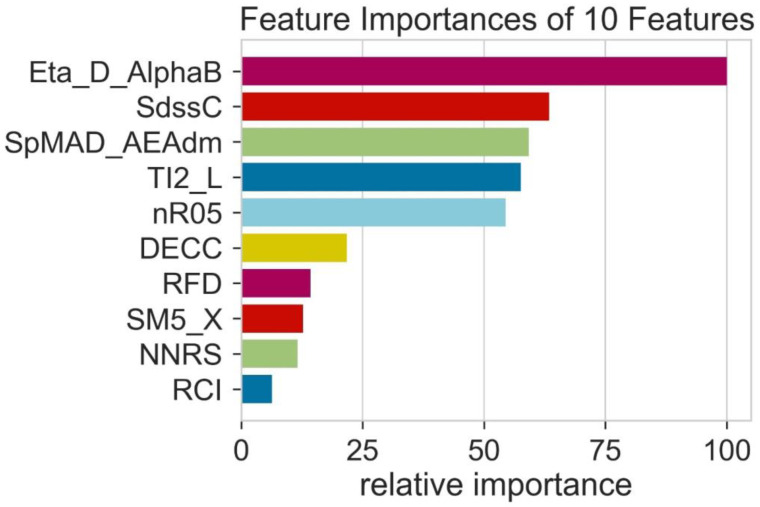
The relative importance of features.

All the molecular descriptors are not able to give the performance value equally during the time of model training [40,41]. Consequently, to evaluate the performance ability of each feature or molecular descriptor, it is important to calculate the relative importance of all the molecular descriptors used. The feature present with a high value of relative importance shows that it can contribute most to the algorithm used in machine learning. Moreover, the feature of high relative importance shows that these are considered helpful for predicting machine learning models. Figure 4 indicates that the molecular descriptor RCI shows the least value of relative importance and its contribution to the algorithms is extremely low. In contrast, ETA-D-AlphaB shows a high value of relative importance, and its contribution is greater than all the other features for the prediction of the machine learning models. The variety of the features shows different relative importance.

**Figure 5:**
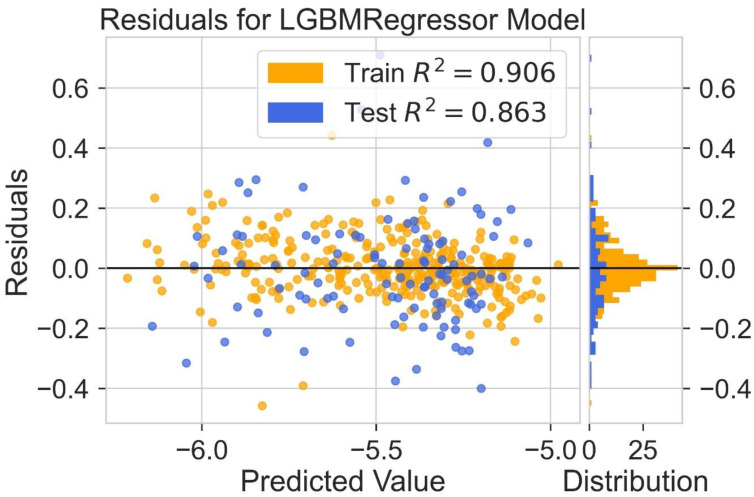
Residual for LGBM regression model.

**Figure 6:**
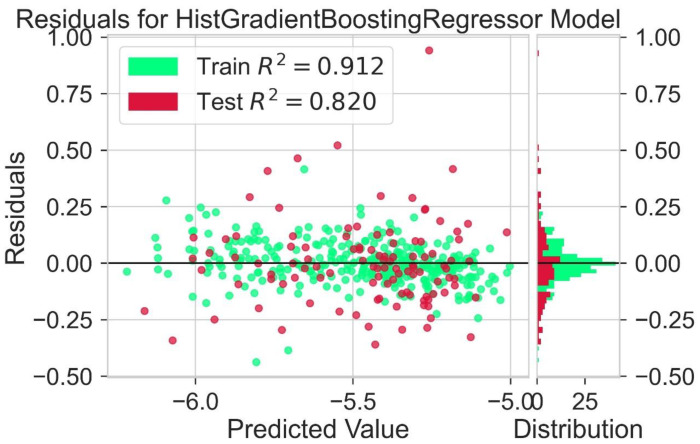
Residuals for Hist gradient booting regression model.

Different models are tested for their predictive capability (Table 2). The light gradient boosting model (LGBM) and Hist gradient boosting are used for further analysis. A residual plot helps to identify problems associated with regression analysis. In the residual plot, the target variable is present on the x-axis and the residuals are on the y-axis. The deviation of the predicted value from the actual value is indicated by the residual values. If the data point is away from the zero line, the prediction value will differ from the actual values. The residual plot for the LGBM regression model is shown in Figure 5. The residual plot for the Hist gradient boosting model is shown in Figure 6. The behavior of LGBM regression models is like that of the Hist gradient boosting regression model. R^2^ is the coefficient of determination for the test and trained value. R^2^ for the test and train residues is not remarkably high: near zero, which is considered accurate. So, the results of both models are good. Dependence on expensive experimental techniques can be decreased by finding accurate results by machine learning models. The more similarities in the predicted and experimental values show that the model or method used was precise and accurate. The easy and fast prediction of results can speed up the design process of new structures of donor materials.

**Figure 7:**
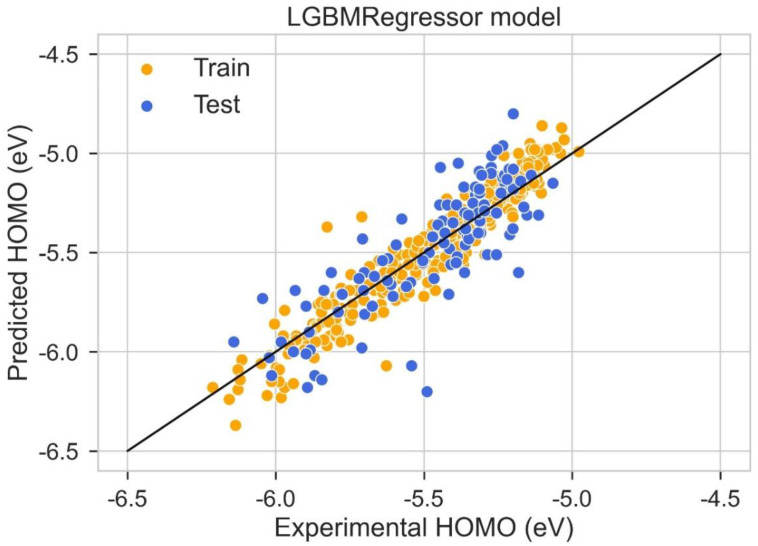
Scatter plot between experimental and predicted HOMO using LGBM regression model.

**Figure 8:**
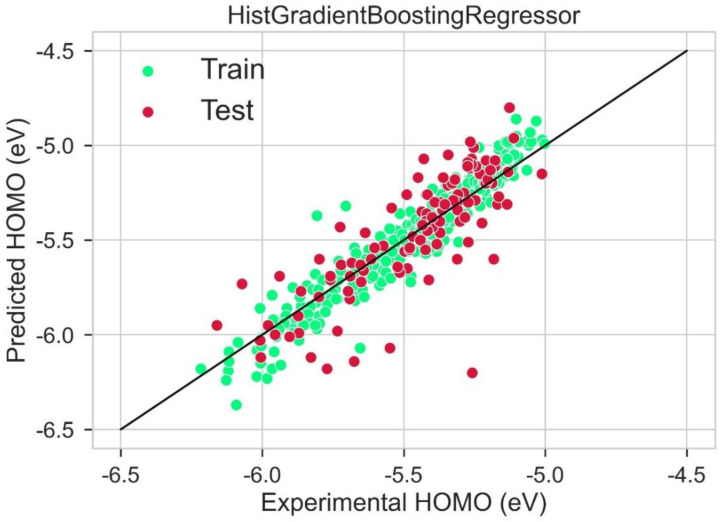
Scatter plot between experimental and predicted HOMO using Hist gradient booting regression.

A scattered plot between the experimental value and expected value of HOMO using the LGBM Regression model and Hist gradient regression model is shown in Figure 7 and Figure 8, respectively. The scatter plot is drawn between the residuals for models and the experimental or predicted value. The majority of values are in the low range, close to zero, which is a clear indication of accurate results. The values for train residues and the values for the test residues are also close to zero. The results show that both LGBM Regression model and the Hist Gradient Boosting Regression model are the best models for regression analysis.

### 2.6. LUMO Prediction

**Figure 9:**
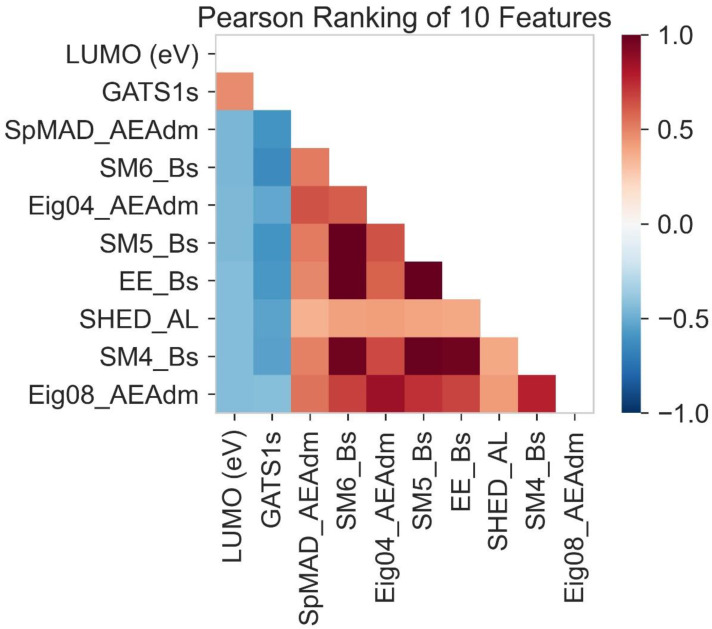
Correlation between LUMO and molecular descriptors.

In Pearson ranking, a correlation between the LUMO and molecular descriptors is determined (Figure 9). The value of the Pearson ranking shows that the value from 0 to +1 shows a positive correlation. The molecular descriptors that fall in the value of 0 to +1 are indicated by red color. There is no correlation at zero point. The molecular descriptors having a blue appearance indicate a negative correlation. The negative correlation ranges from 0 to −1.

GATS1s is the molecular descriptor (Table 3) presented on the y-axis. It indicates a positive correlation with LUMO lying on the x-axis, while the other molecular descriptors present on the y-axis show a negative correlation because their blue color indicates that the values of these molecular descriptors must lie between 0 and −1. In contrast, SPMAD-AEAdm, Eig04_EA(dm), EE_B(s), SM4_B(s), SM5_B(s), SM6_B(s), SHED-AL and Eig08_EA(dm) are the molecular descriptors present on the x-axis. These indicate a positive correlation with the LUMO present on the y-axis (red in color).

**Figure 10:**
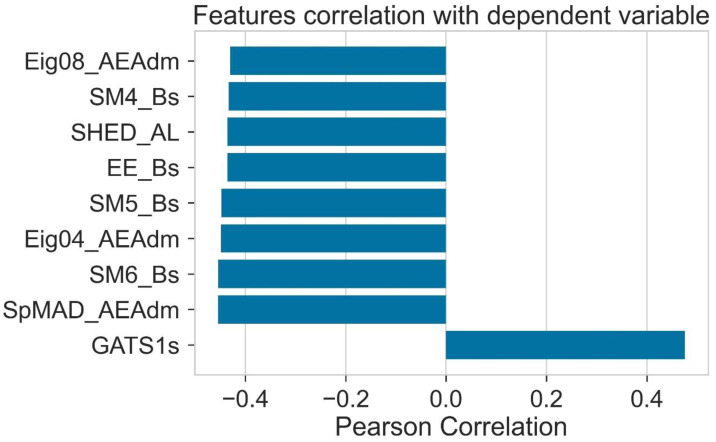
Correlation of features with LUMO.

A source of input is considered a calculated molecular descriptor for the model training. The chemistry of donor molecules is represented by the help of molecular descriptors. Figure 10 shows that molecular descriptors such as SPMAD-AEAdm, Eig04_EA(dm), EE_B(s), SM4_B(s), SM5_B(s), Eig08_EA(dm), SM6_B(s), and Eig08_EA(dm) indicate the negative dependent Pearson correlation. This negative correlation is determined by noting that these molecular descriptors lie between 0 and _1. In LUMO’s case, only one molecular descriptor, GATS1s, shows a positive dependent correlation, with a value from 0 to +1.

**Figure 11:**
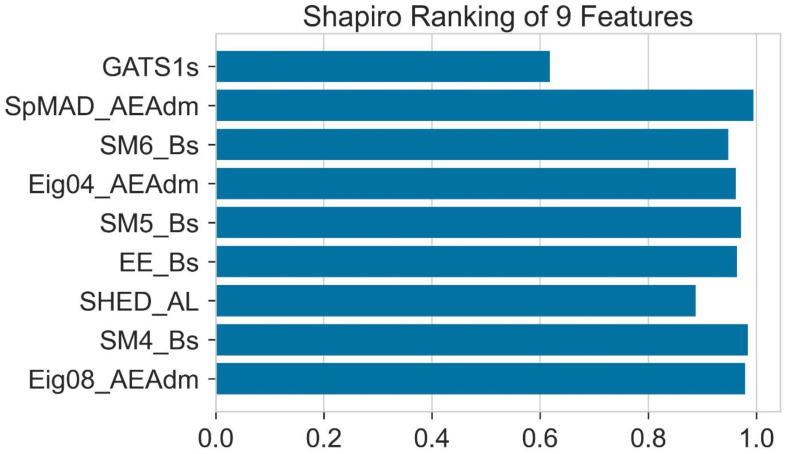
The normality of all the features analyzed by Shapiro test.

To find the normality of the distribution of molecular descriptors, Shapiro ranking deals with a single molecular descriptor one at a time. In Figure 11, GATS1s shows the least normality according to Shapiro ranking. On the other hand, SPMAD-AEAdm and SM4-BS show the greatest normality according to this ranking.

**Figure 12:**
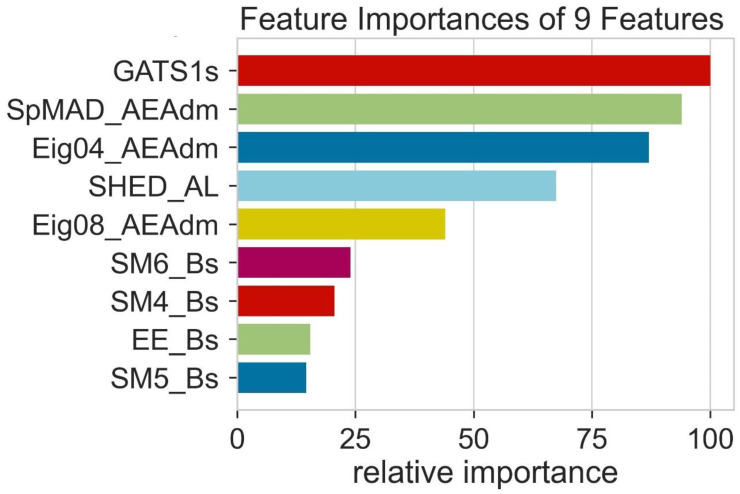
The relative importance of features.

The relative importance of features tells us about the performing ability of different molecular descriptors. During the training of the model, all the molecular descriptors present are not able to perform at an equal level. It is important to calculate the relative importance of all the molecular descriptors to check the performance ability of each. So, the relative importance of features helps to evaluate the performing ability of different molecular descriptors. The molecular descriptor whose relative importance is high shows that it can be used mostly for the prediction of results. Additionally, the feature with the highest value of relative performance among all the features is considered helpful in training algorithms used in machine learning. Figure 12 shows that the molecular descriptor SM5-Bs shows the least value of relative importance and its contribution to the algorithms is extremely low. On the other hand, the molecular descriptors GATS1s, SPMAD-AEAdm, Eigo4-AEAdm and SHED-AL show high values of relative importance. The variety of features shows different relative importance.

**Figure 13:**
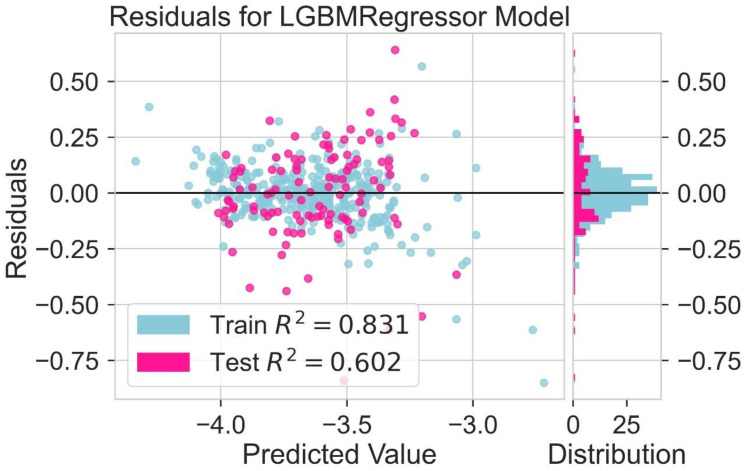
Residual for LGBM regression model for LUMO prediction.

**Figure 14:**
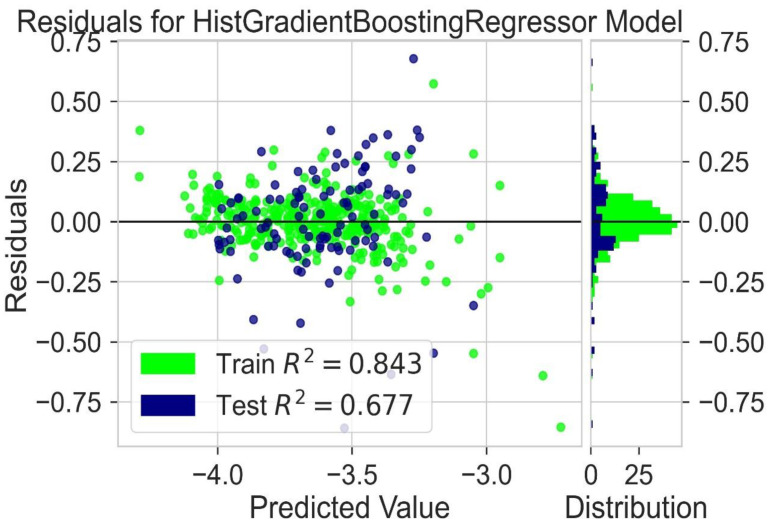
Residuals for Hist gradient booting regression model for LUMO prediction.

A variety of regression models are used for the prediction of results [42]. R^2^ values are given in Table 4. LGBM and Hist gradient boosting models consider the best working models for the prediction of LUMO. An analysis of different molecular descriptors is carried out by using these regression models. A residual plot is used to identify problems with regression analysis. In the residual plot, the relationship between the test value and the train value is predicted. The target variable is present on the x-axis, and the residuals are on the y-axis. If the value of train data is near the value of test data, then the chances of accurate results increase. If the values of the test and train data are not near each other or near the zero line, it indicates that the prediction value will differ further from the actual values. The residual plot for the LGBM regression model is shown in Figure 13. The residual plot for the Hist gradient boosting model is shown in Figure 14. The obtained results show that the behavior of LGBM regression models is like that of the Hist Gradient Boosting regression model. The coefficient of determination for the test and trained value is indicated by the symbol R^2^. These regression models show that the value of R^2^ is near zero. So, the results of both models are considered good enough. By using machine learning models, accurate results can be achieved. This is helpful to avoid expensive experimental techniques.

**Figure 15:**
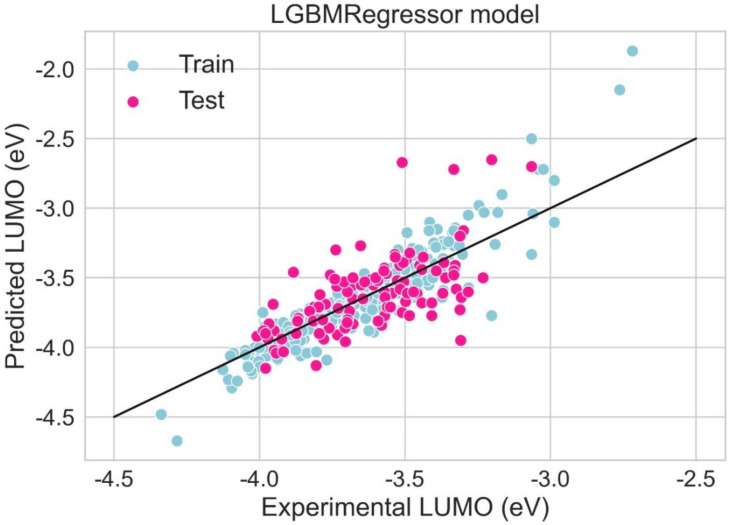
Scatter plot between experimental and predicted HOMO using LGBM regression model.

**Figure 16:**
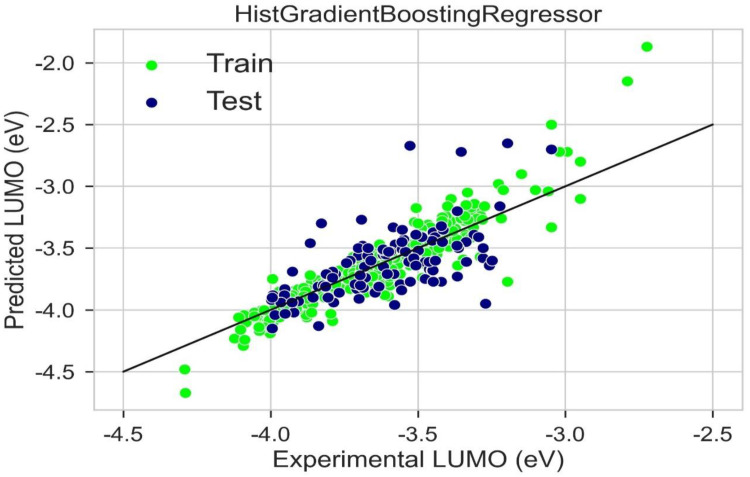
Scatter plot between experimental and predicted LUMO using Hist gradient booting.

A scattered plot between the experimental value and expected value of LUMO using the LGBM regression model and Hist gradient regression model is shown in Figure 15 and Figure 16, respectively. The scatter plot is drawn between the residuals for the models and the experimentally predicted value. It is mostly used to find problems with regression models. For data points above the line, residuals are positive. For the data points below the line, the residuals are negative. The closer the value of the data points to 0, the more accurate it is for results. The scatter plot of LGBM and Hist gradient regression models shows that most of the values lie in the low range, near the value of zero, indicating accurate results.

### 2.7. Database Mining

**Figure 17:**
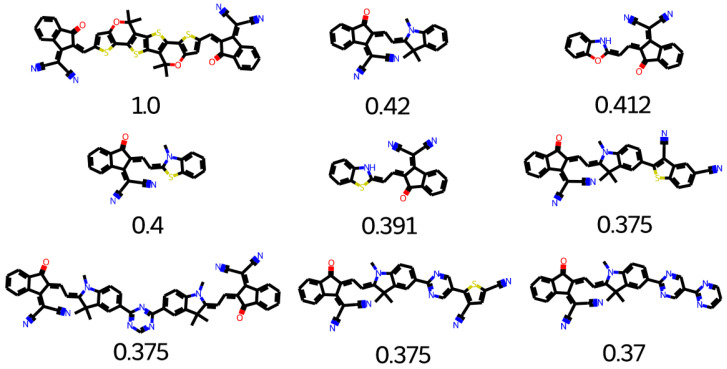
Top search hit for O4TIC.

**Figure 18:**
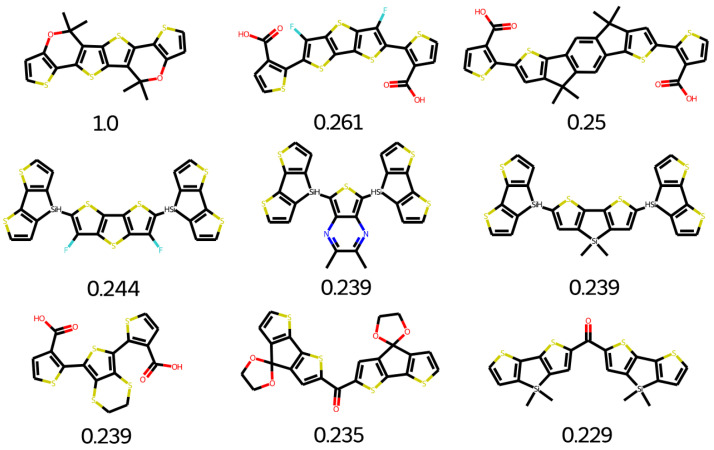
Top search hit for middle O4TIC.

The Clean Energy Project (CEP) is a database that contains thousands of organic molecules. These molecules can be used for various photovoltaics applications [43,44]. A similarity analysis is performed to find suitable building units. O4TIC is a low-band gap molecule. O4TIC contains a carbon–oxygen bridged-type ladder with strong electron-donating capability with the oxygen atoms conjugation effect. The further band gap of the molecule is decreased with the attachment of a more electron-rich group instead of the central phenyl group, which increases the donating capability of the molecule [43,45]. The linear side increases the crystallinity, which in turn increases the mobility of the electrons. The top search hits for O4TIC references are given in Figure 17. The building blocks found are not overly similar to O4TIC; however, most are suitable for the design of polymers for organic solar cells. The top search hits for middle O4TIC are given in Figure 18. All the structures are unique and possible to synthesize.

**Figure 19:**
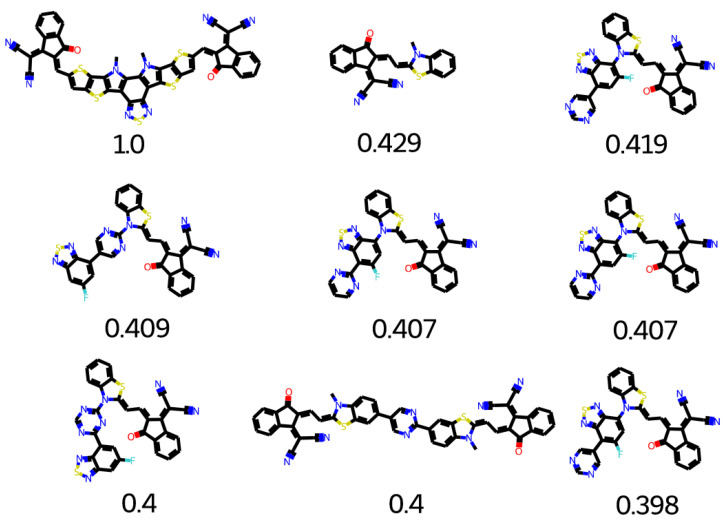
Top search hit for Y5.

**Figure 20:**
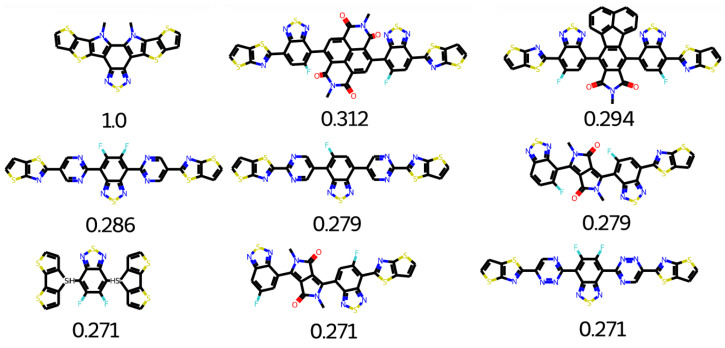
Top search hit for Y5 middle.

The top search hits for Y5 are given in Figure 19. Many groups can be used for polymer designing. After a minor structural modification, other groups can also be useful candidates. The top search hits for Y5 middle are given in Figure 20. Molecular core of Y5 is used as an electron deficient group. Y5 core structure is considered as high performance (NFA) non-fullerene acceptor. Y5 can be applied to both inverted and conventional OPV devices because of versatility of Y5. OSCs based on NFA can achieve longer device life-time with greater photochemical and thermal stability [46]. The combination of Y5 electron deficient with five different donor polymers could lead to enhanced efficiency [47].

The organic building units are varied because various positions are available to add or connect plenty of heteroatoms [48,49,50,51]. This is carried out to produce or synthesize countless organic molecules that are better in their characteristics than the previous ones. To design new polymers or organic semiconductors for organic photodetectors, electron-deficient and electron-rich groups can be used. Hundreds of building blocks can be selected based on the addition of terminal groups and the availability of the position for alkyl chains. Many organic semiconductor materials can be designed by connecting new building units. A suitable combination of electron-rich and electron-deficient results in the formation of an electron hole, which leads to an increase in conjugation [52,53,54,55].

## 3. Methodology

### 3.1. Dataset

The data for machine learning were collected from research papers. The volume of data was enough for good machine learning models. The data are based on energy levels and photovoltaic parameters. The performance of the machine learning model strongly depends on the quality and quantity of the data [56].

### 3.2. Molecular Descriptor Calculation

Several types of molecular descriptors of molecules were calculated using Dragon software [57]. About 4000 descriptors were generated. The best descriptors were shortlisted using univariate regression. These descriptors were used for training machine learning models.

### 3.3. Training the Model

We have imported the necessary packages of Python such as Scikit-learn, Pandas, Scipy, Numpy, Seaborn, and Matplotlib. These packages are necessary for data visualization and analysis. The calculated descriptors and target properties in comma-separated value (CSV) files were imported with the help of the Pandas module.

### 3.4. Similarity Analysis

A similarity analysis was performed using RDKit [58]. The similarity analysis is a straightforward method to find the similarities between reference structure and structure in the database. For this purpose, pharmacophores, distances, fingerprints, etc., can be used. In our work, Tanimoto similarity was used. For this purpose, ECFP4 fingerprints were selected.

## 4. Conclusions

In summary, data on large photovoltaic properties were collected from already reported experimental studies and subsequently utilized to train machine learning models. Among the multiple trained models, the LGBM regression model and Hist gradient booting regression model demonstrated the best predictive capability. Moreover, HOMO and LUMO energy levels were successfully predicted. The results revealed that good consistency was obtained between experimental outcomes and model predictions. In addition, Pearson correlation and Shapiro ranking was applied to demonstrate the correlation between different parameters. Furthermore, a similarity analysis was performed to find the similarities between reference structure and structure in the database. The reliability of our designed approach was also verified by mining the photovoltaic database to search for new building units. This indicates that machine learning is a powerful approach to predict the properties of photodetectors, which can facilitate their rapid development in various fields. Fast screening or searching of new building units with minimal computational costs could significantly reduce experimentation (trial and error methods) costs by narrowing down the search for potential candidates.

## Figures and Tables

**Table 1:** Molecular descriptors with respective categories and descriptions.

No	Molecular Descriptor	Category	Description
1	SM5_X	2D matrix-based descriptors	Spectral moment of order 5 from chi matrix
2	RCI	Ring descriptors	Ring complexity index
3	nR05	Ring descriptors	Number of 5-membered rings
4	RFD	Ring descriptors	Ring fusion density
5	NNRS	Ring descriptors	Normalized number of ring systems
6	DECC	Topological indice	Eccentric
7	ETA-D-AlphaB		Eta delta alpha b index
8	SdssC	Atom-type E-state indices	Sum of dssC E-states
9	SpAD_AEA(dm)	Edge adjacency indices	Spectral absolute deviation from augmented edge adjacency mat. weighted by the dipole moment
10	TI2-LN	2D matrix-based descriptors	Second Mohar index from Laplace matrix

**Table 2:** R^2^, mean absolute error (MAE) and root mean square error (RMSE) values of various models for HOMO prediction.

Model	Train R^2^	Test R^2^	Train MAE (eV)	Test MAE (eV)	Train RMSE (eV)	Test RMSE (eV)
Hist Gradient Boosting Regressor	0.912	0.820	0.136	0.146	0.163	0.176
LGBM Regressor	0.906	0.863	0.137	0.142	0.165	0.172
Random Forest Regressor	0.853	0.801	0.144	0.148	0.174	0.180
Decision Tree Regressor	0.752	0.683	0.150	0.155	0.183	0.193
Extra Trees Regressor	0.723	0.652	0.152	0.159	0.189	0.194
AdaBoost Regressor	0.623	0.560	0.161	0.172	0.1950	0.239
K-Neighbors Regressor	0.620	0.564	0.161	0.171	0.1950	0.237
Linear Regression	0.610	0.550	0.162	0.173	0.1960	0.243

**Table 3:** Molecular descriptors with respective categories and descriptions.

No	Molecular Descriptor	Category	Description
1	SpAD_AEA(dm)	Edge adjacency indices	Spectral absolute deviation from augmented edge mat. weighted by dipole moment
2	GATS1s	2D autocorrelations	Geary autocorrelation of lag 1 weighted by I-state
3	Eig04_EA(dm)	Edge adjacency indices	Eigenvalue n. 4 from edge adjacency mat. weighted by dipole Moment
4	EE_B(s)	2D matrix-based descriptor	Estrada-like index (log function) from Burden matrix weighted by I-State
5	SM4_B(s)	2D matrix-based descriptors	Spectral moment of order 4 from Burden matrix by I-State
6	SM5_B(s)	2D matrix-based descriptors	Spectral moment of order 5 from Burden matrix by I-State
7	SM6_B(s)	2D matrix-based descriptors	Spectral moment of order 6 from Burden matrix by I-State
8	Eig08_EA(dm)	Edge adjacency indices	Eigenvalue n. 8 from edge adjacency mat. weighted by dipole Moment
9	SHED-AL		SHED Acceptor Lipophilic

**Table 4:** R^2^, mean absolute error (MAE) and root mean square error (RMSE) values for LUMO prediction.

Model	Train R^2^	Test R^2^	Train MAE (eV)	Test MAE (eV)	Train RMSE (eV)	Test RMSE (eV)
Hist Gradient Boosting Regressor	0.843	0.667	0.070	0.074	0.084	0.089
LGBM Regressor	0.831	0.602	0.071	0.075	0.085	0.090
Random Forest Regressor	0.820	0.601	0.072	0.076	0.087	0.092
Decision Tree Regressor	0.732	0.583	0.075	0.078	0.093	0.097
Extra Trees Regressor	0.723	0.570	0.076	0.080	0.095	0.097
AdaBoost Regressor	0.652	0.540	0.081	0.086	0.098	0.120
Linear Regression	0.612	0.504	0.082	0.087	0.099	0.121
K-Neighbors Regressor	0.610	0.520	0.081	0.087	0.098	0.122

## Data Availability

Associated data used in this paper can be obtained from the corresponding author upon reasonable request.
